# Custom-Made Synthetic Scaffolds for Bone Reconstruction: A Retrospective, Multicenter Clinical Study on 15 Patients

**DOI:** 10.1155/2016/5862586

**Published:** 2016-12-14

**Authors:** Fabrizia Luongo, Francesco Guido Mangano, Aldo Macchi, Giuseppe Luongo, Carlo Mangano

**Affiliations:** ^1^Private Practice, 00193 Rome, Italy; ^2^Department of Surgical and Morphological Science, Dental School, Insubria University, 21100 Varese, Italy; ^3^Department of Oral and Maxillofacial Surgery, Federico II University, 80131 Naples, Italy; ^4^Department of Dental Sciences, Vita Salute San Raffaele University, 20132 Milan, Italy

## Abstract

*Purpose*. To present a computer-assisted-design/computer-assisted-manufacturing (CAD/CAM) technique for the design, fabrication, and clinical application of custom-made synthetic scaffolds, for alveolar ridge augmentation.* Methods*. The CAD/CAM procedure consisted of (1) virtual planning/design of the custom-made scaffold; (2) milling of the scaffold into the exact size/shape from a preformed synthetic bone block; (3) reconstructive surgery. The main clinical/radiographic outcomes were vertical/horizontal bone gain, any biological complication, and implant survival.* Results*. Fifteen patients were selected who had been treated with a custom-made synthetic scaffold for ridge augmentation. The scaffolds closely matched the shape of the defects: this reduced the operation time and contributed to good healing. A few patients experienced biological complications, such as pain/swelling (2/15: 13.3%) and exposure of the scaffold (3/15: 20.0%); one of these had infection and complete graft loss. In all other patients, 8 months after reconstruction, a well-integrated newly formed bone was clinically available, and the radiographic evaluation revealed a mean vertical and horizontal bone gain of 2.1 ± 0.9 mm and 3.0 ± 1.0 mm, respectively. Fourteen implants were placed and restored with single crowns. The implant survival rate was 100%.* Conclusions*. Although positive outcomes have been found with custom-made synthetic scaffolds in alveolar ridge augmentation, further studies are needed to validate this technique.

## 1. Introduction

The rehabilitation of partial and total edentulism using dental implants is today considered a successful treatment procedure, with very high survival and success rates [1–3].

However, it is frequently the case that the available bone is not sufficient for a direct implant insertion. Maxillary/mandibular bone defects are rather frequent, as a result of different processes such as tooth loss, periodontal disease, trauma, and tumours [4, 5]. In all these cases, the placement of an implant in the correct three-dimensional (3D) position can be unachievable, and therefore the complete restoration of the function and aesthetics of the patient with an implant-supported restoration is impossible [4, 5].

Even if the literature has reported successful long-term results with the use of short [6, 7] and tilted implants [8] in regions with high bone resorption, the best option from a functional and aesthetic point of view remains to reconstruct the normal bone volume of the dentoalveolar process [5].

Many different techniques have been developed in order to reconstruct alveolar ridge defects and therefore allow the correct 3D insertion of dental implants [9–14]. Among the different procedures that can be used to regenerate bone defects are guided bone regeneration (GBR) with membranes [9], the application of onlay/inlay bone blocks [10, 11], maxillary sinus augmentation [12], and the use of bone distraction [13] or the split-crest technique [14].

Although all these surgical techniques can be successful in regenerating bone, the incidence of failures and problems that occur during these augmentation procedures is rather high [4, 5, 9–13]. In fact, these techniques are complex and require skill and experience from the operator; the operating time can be lengthy with major discomfort for the patient, and the risk of complications can be high [4–6, 9–13].

The most predictable material for regeneration of the dentoalveolar process is autogenous bone, due to its peculiar properties: it is in fact osteoinductive, osteoconductive, and inherently osteogenic [15]. However, this material requires harvesting from other anatomical sites (intra- or extra-orally); in order to reduce patients' discomfort and the complications related to the harvesting procedures, several bone substitutes have been introduced, such as allografts, xenografts, and, more recently, alloplasts [15, 16].

An ideal bone substitute should have excellent osteoconductivity: in fact, it should be capable of guiding the growth and proliferation of osteoblasts onto its surface [16, 17]. Ideally, it should be osteoinductive too: it should stimulate the differentiation of mesenchymal stem cells into the osteoblastic lineage [17, 18].

Recently, the use of scaffolds made of synthetic alloplastic materials has gained attention [19]. Since the crystalline phase of natural bone is hydroxyapatite, synthetic ceramics are now frequently used as bone substitutes [19].

An ideal bone substitute should easily fit into the receiving site, with a perfect shape, obtained with simple procedures [20, 21]. With the conventional augmentation procedures, the blocks of different materials have to be manually adapted during the surgery [20–24]. This procedure is time-consuming and is highly dependent on the clinician's skill and experience. The complexity of these procedures can lead to the modification of the scaffold properties [20–24]; this can result in a gap between the scaffold and the natural bone that needs to be filled with particulate grafts.

With the development of new digital technologies, it is now possible to analyse the bone defects in 3D and to customize bone grafts that fit perfectly into the receiving site [20–22]. In fact, the recent improvements in computer-guided technologies provide clinicians with the possibility of evaluating the size and shape of the bone defect in 3D, before the surgery, with the aid of a cone beam computed tomography (CBCT) examination [20–25]. CBCT files can be transferred to specific reconstruction software, where a 3D model of the maxilla/mandible of the patient can be easily obtained [25, 26]. Finally, a custom-made bone graft can be designed directly on this 3D model, using powerful computer-assisted-design (CAD) software [20, 21, 25, 26]. The custom-made bone graft is then milled with a computer-numeric-control (CNC) machine, in the selected material (allograft, xenograft, or alloplast), according to the file received from the 3D planning made by the surgeon [20, 21, 25, 26]. This custom-made bone graft will be easily adapted in the surgical site, with high accuracy: this approach can facilitate surgery, reducing the operative time and discomfort of the patient [20, 21, 25, 26].

The aim of the present retrospective clinical study is to report on the clinical and radiographic outcomes of bone reconstruction procedures performed with custom-made synthetic bone grafts, in three different clinical centres.

## 2. Materials and Methods

### 2.1. Inclusion and Exclusion Criteria

The data of the patients considered for inclusion in the present retrospective clinical study came from the dental records of two different private clinics (located, resp., in Gravedona, Como, and Rome, Italy) and from the database of the dental clinic of the Insubria University (Varese, Italy). Inclusion criteria for this retrospective study were patients with a single tooth gap in the anterior/posterior maxilla or mandible, with a residual bone width of 3-4 mm, associated with a 3-wall bone defect. All these patients needed bone augmentation, prior to allowing the proper placement of dental implants and the fabrication of a functional and aesthetically acceptable implant-supported restoration. Exclusion criteria were the presence of active periodontal disease or active infection at the surgical site; poor oral hygiene or hygienic compliance; heavy cigarette smoking (>15 cigarettes/day); treatment with bisphosphonates (intraoral and/or intravenous) and any medical/general condition that could contraindicate surgery (such as immunocompromised status, uncontrolled diabetes, chemotherapy/radiotherapy of the head/neck, hepatitis, and HIV). All patients received full information about the risks related to the treatment procedure and therefore signed an informed consent form. The local ethics committee approved the present study, which was conducted in accordance with the Declaration of Helsinki on experimental studies involving human subjects, as revised in 2008.

### 2.2. Data Acquisition and Elaboration

**Figure 1:**
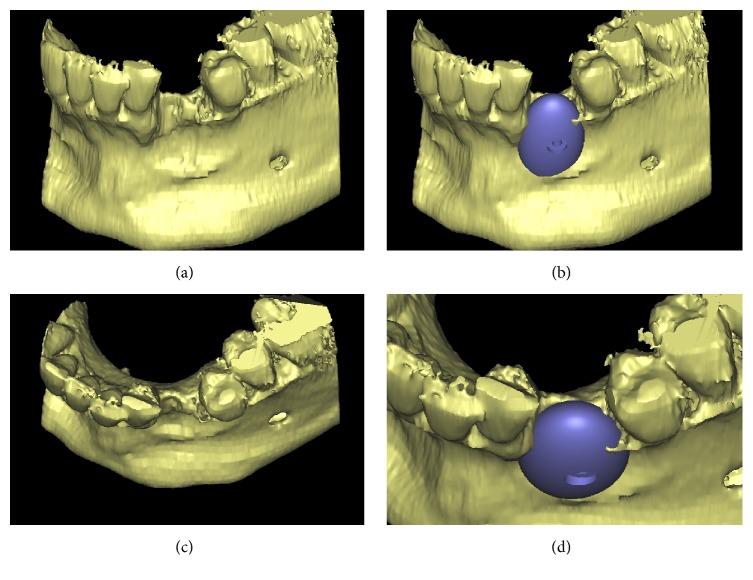
Virtual 3D model of the deficient ridge and first drawing of the customized scaffold (Mimics®, Materialise. Leuven, Belgium): (a) frontal view of the ridge without the customized scaffold; (b) frontal view of the ridge with the customized scaffold; (c) occlusal view of the ridge without the customized scaffold; (d) occlusal view of the ridge with the customized scaffold.

**Figure 2:**
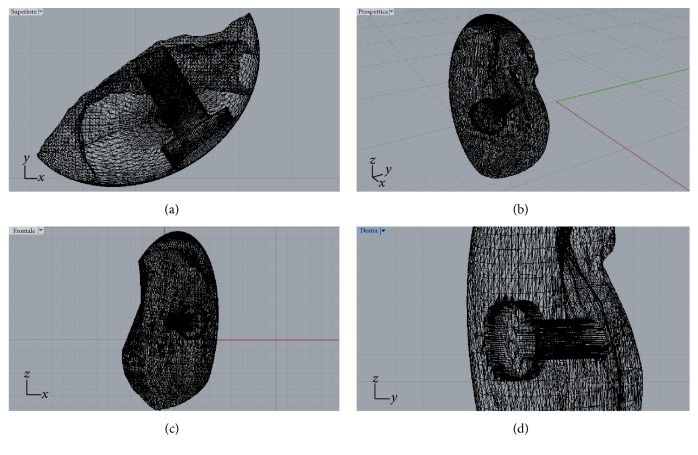
The customized synthetic scaffold was designed with a hole in its centre to allow the placement of a fixation screw (Rhino®, Robert McNeel & Associates, Seattle, WA, USA): (a-b-c-d) different views of the scaffold design.

**Figure 3:**
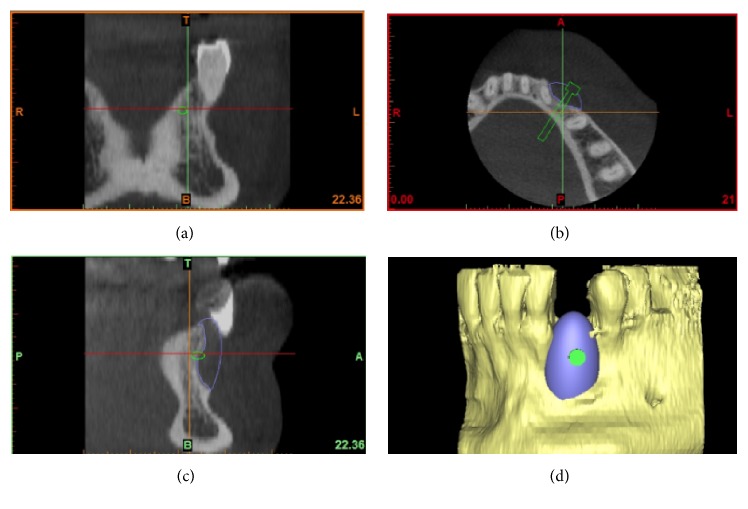
The proper size/shape of the customized scaffold was verified and the adaptation to the bone defect and the congruence with the bony walls was perfectioned (Mimics®, Materialise. Leuven, Belgium): (a) frontal view; (b) axial view; (c) lateral view; (d) 3D reconstruction.

A careful assessment of the oral hard and soft tissues was performed on each patient. Panoramic and periapical radiographs were the primary investigation; after that, each patient underwent a CBCT examination, with a modern scanner (CS 9300, Carestream Health, Rochester, NY, USA). Different fields-of-view (FOV) were selected, according to the clinical indications. CBCT datasets of the partially edentulous ridges, acquired in the DICOM (Digital Imaging and Communications in Medicine) format, were then uploaded onto a proprietary 3D reconstruction software (Mimics, Materialise, Leuven, Belgium) where bone segmentation was carefully performed, using thresholding tools. A virtual model of the partially edentulous ridge was therefore obtained, where the bone defect was clearly visible, and a first drawing of the scaffold could be performed (Figures 1(a), 1(b), 1(c), and 1(d)); both these models were saved as a solid-to-layer (STL) files and then transferred to another proprietary CAD software (Rhino, Robert McNeel & Associates, Seattle, WA, USA). The aforementioned software allowed the completion of the 3D design of the anatomically-shaped, custom-made scaffold. This scaffold was designed with a hole in its centre (Figures 2(a), 2(b), 2(c), and 2(d)) to allow the placement of a fixation screw, saved again as an STL file and reimported into the Mimics software. Here, the correct size/shape of the scaffold was verified; in addition, the adaptation to the bone defect and the congruence with the bony walls was perfected (Figures 3(a), 3(b), 3(c), and 3(d)).

### 2.3. Fabrication of the Customized Synthetic Scaffolds

**Figure 4:**
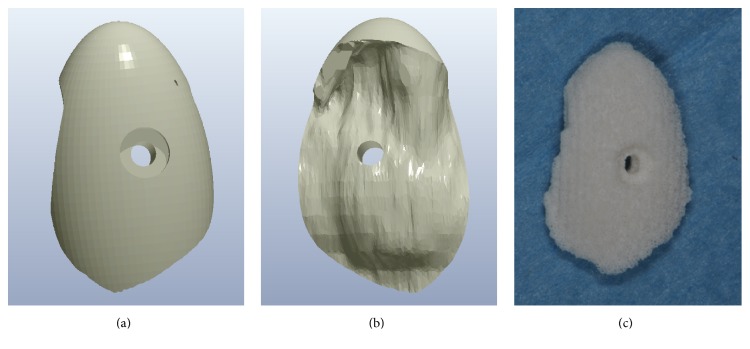
A block of synthetic micromacroporous biphasic calcium-phosphate (BCP), consisting of 70% beta-tricalcium-phosphate and 30% hydroxyapatite (BTK®, Dueville, Vicenza, Italy), was placed into the CNC milling machine (DWX-51®, Roland DG Mid Europe, Acquaviva Picena, AP, Italy) and milled into the size/shape of the 3D virtual model, so that a customised synthetic BCP scaffold was fabricated: (a) buccal aspect of the scaffold design; (b) lingual aspect of the scaffold design; (c) the milled customized scaffold ready for clinical use.

The 3D virtual model of the custom-made scaffold was imported into a proprietary computer-assisted-manufacturing (CAM) software (Hyperdent, Open Mind Technologies AG, Wessling, Germany) and used to generate a set of tool-paths for fabrication by a proprietary CNC milling machine (DWX-51, Roland DG Mid Europe, Acquaviva Picena, AP, Italy). A synthetic micro-macroporous biphasic calcium-phosphate (BCP) block, consisting of 70% beta-tricalcium-phosphate and 30% hydroxyapatite (BTK, Dueville, Vicenza, Italy) was selected as the material of choice for the fabrication of the custom-made scaffolds. The block was therefore placed into the CNC milling machine, and milled into the size/shape of the 3D virtual model, so that a custom-made synthetic BCP scaffold was fabricated (Figures 4(a), 4(b), and 4(c)). The custom-made synthetic block was sterilised and it was therefore ready for clinical use.

### 2.4. Bone Reconstruction

**Figure 5:**
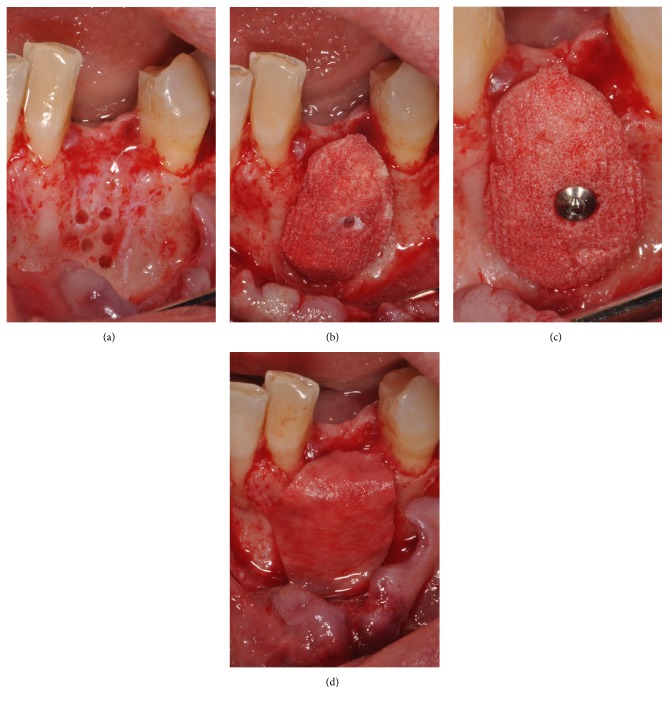
Bone reconstruction: (a) a full-thickness flap was elevated in order to fully expose the bone defect, and a series of little perforations, 1-2 mm deep, were made on the defect walls, in order to increase the amount of bleeding at the surgical site; (b) the customized synthetic scaffold was placed in position; (c) the scaffold was fixed to the remaining wall by means of a titanium mini-screw, positioned through the predetermined hole; (d) an absorbable collagen membrane was placed, in order to protect the scaffold.

After the injection of local anaesthesia, a full-thickness flap was elevated. The main incision (which was slightly palatal/lingual to the bone crest) was connected with two deep, lateral releasing incisions, in order to properly view the area of the defect. The bony architecture, and consequently the bone defect, was fully exposed. A series of small perforations, 1-2 mm deep, were made on the defect walls, in order to increase the amount of bleeding at the surgical site (Figure 5(a)). The custom-made, synthetic scaffold was then placed into position (Figure 5(b)) and fixed to the remaining wall by means of a titanium mini-screw, positioned through the predetermined hole (Figure 5(c)). Care was taken not to break the synthetic scaffold during fixation. An absorbable collagen membrane was used in order to protect the scaffold (Figure 5(d)). Before suturing, the mucoperiosteal flap was widely mobilised by means of a series of horizontal releasing incisions directly on the periosteum. The widely mobilised flap was thereby sutured in position, without any tension, by means of absorbable sutures. All patients were prescribed oral antibiotics, amoxicillin plus clavulanic acid, 1 gr every 12 hrs for an entire week. Postoperative pain was controlled with analgesics, 600 mg of Ibuprofen every 12 hours for the first 2/3 days. Finally, chlorexidine 0.12% mouth rinses were prescribed, 2/3 times a day for one week.

### 2.5. Implant Placement and Prosthetic Procedures

**Figure 6:**
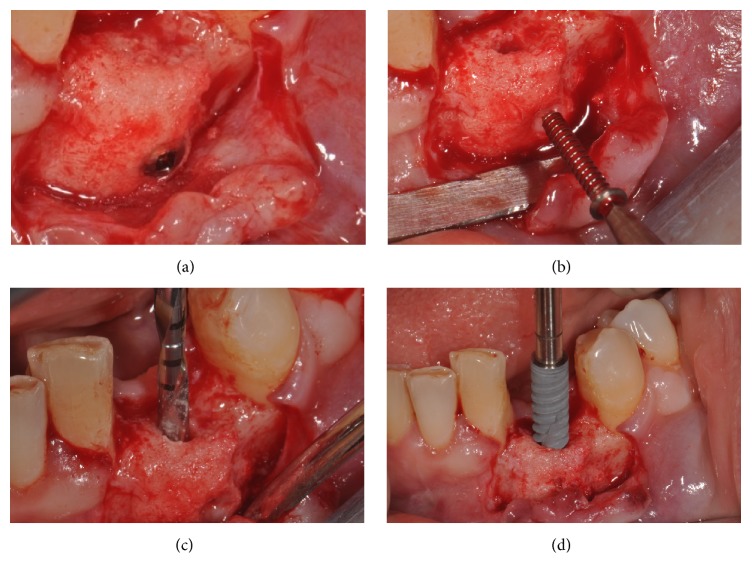
After 8 months from the regenerative surgery, the implant was placed: (a) after the elevation of a full-thickness flap, the regenerated site showed an increased bone thickness with a considerable amount of new, clinically well-integrated bone; (b) the mini-screw used for fixation was removed; (c) the preparation of the surgical site was performed with drills of increasing diameter; (d) a 3.5 diameter × 13 mm length implant (NobelActive®, Nobel Biocare, Kloten, Switzerland) was placed in the regenerated site.

**Figure 7:**
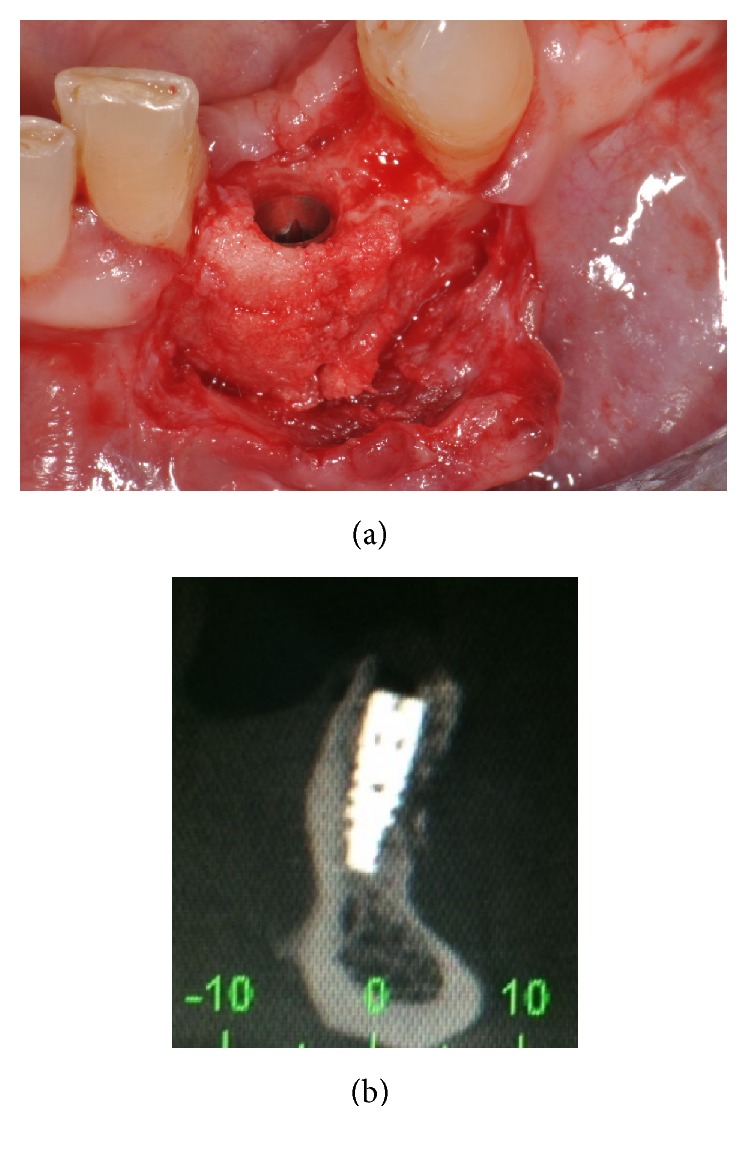
The implant was located in the perfect 3D position: (a) it was placed in a clinically well-integrated, regenerated bone; (b) as confirmed by the CBCT control examination.

**Figure 8:**
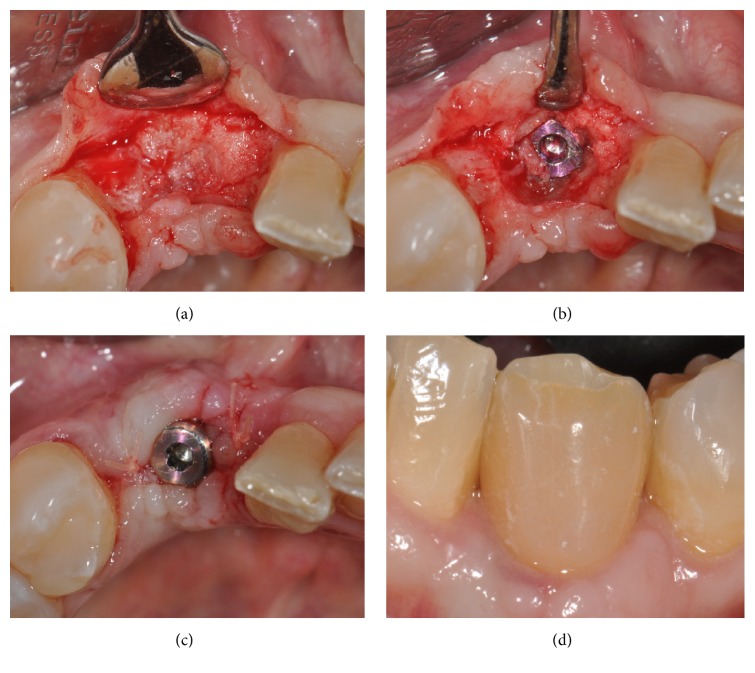
After being submerged for a period of 2-3 months, the implant was uncovered and a prosthetic restoration was placed: (a) a full-thickness surgical flap was raised; (b) the implant was uncovered; (c) the cover cap was replaced by a transmucosal healing abutment and interrupted sutures were performed; (d) two weeks later, the provisional crown was placed.

**Figure 9:**
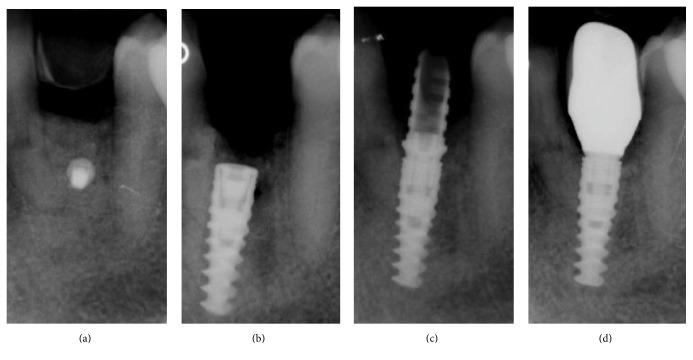
Radiographic history of the case: (a) periapical rx taken immediately after the reconstructive procedure; (b) periapical rx taken immediately after implant placement; (c) the implant during the provisional phase, 4 months after placement of the fixture; (d) final rx control of the definitive crown, 3 years after implant placement.

The sutures were removed 8–12 days after the surgery. An undisturbed 8-month healing protocol was strictly followed by all patients. During this healing period, the patients were not allowed to use any temporary removable partial denture, in order to avoid any possible compression on the regenerated area. Eight months after the regenerative surgery, the surgical site was exposed again, through the elevation of a full-thickness flap. The mini-screw used for fixation was removed, and the regenerated site showed an increased bone thickness with a considerable amount of new, well-integrated bone (Figures 6(a) and 6(b)). The surgical site was then prepared with a sequence of drills of ascending diameter and a screw-shaped dental implant was inserted (Figures 6(c) and 6(d)). The implant was located in the perfect 3D position, in a clinically well-integrated, regenerated bone (Figure 7(a)), as confirmed by the CBCT control examination (Figure 7(b)). Sutures were performed and the implants were left submerged for a period of 2-3 months. After this short healing period, the implant was uncovered: for the third time, a full-thickness surgical flap was raised (Figure 8(a)); the cover cap (Figure 8(b)) was replaced by a transmucosal healing abutment (Figure 8(c)) and interrupted sutures were performed. Two weeks later, impressions were taken for the fabrication of a provisional resin restoration (Figure 8(d)). This temporary restoration remained in situ for a period of 2 months; then it was replaced by the definitive metal-ceramic or full-ceramic crown (Figures 9(a), 9(b), 9(c), and 9(d)). All temporary and definitive restorations were single crowns, cemented with a temporary zinc-eugenol cement (TempBond®, Kerr, Orange County, CA, USA). Occlusion was carefully checked intraorally, using articulating papers. All patients were placed on a 6-month maintenance program.

### 2.6. Clinical and Radiographic Outcomes

The main clinical and radiographic outcomes of the present study were vertical/horizontal bone gain, any biological complication occurring after bone reconstruction, and implant survival. All these outcomes were carefully checked, in all patients, 6 months after implant placement and at each subsequent follow-up appointment.

#### 2.6.1. Vertical and Horizontal Bone Gain

The vertical and horizontal dimensions of the alveolar ridge were measured in the CBCT sections, before and 8 months after the reconstructive surgery, in mm. Before reconstructive surgery, one first linear measure was taken at the future implant location. Eight months later, immediately after the placement of the implants, the same measures were repeated at the same location. These second measures were registered; then the vertical and horizontal bone gain were calculated as the difference between the second and first measurements.

#### 2.6.2. Implant Survival

At each follow-up control appointment, the single crowns were removed and the stability of the implants was tested. An implant was classified as a surviving implant if still in function, without any problem, at the last follow-up control. Conversely, absence of osseointegration with implant mobility, progressive marginal bone loss due to bacterial tissue invasion (peri-implantitis), and severe marginal bone loss in the absence of symptoms/signs of infection were the conditions in which an implant was considered failed and had to be removed.

#### 2.6.3. Biological Complications

The biological complications were divided into early complications (i.e., complications that occurred* before* the implant placement, such as pain or discomfort after reconstructive surgery, edema, swelling, intra- or extra-oral contusion, early scaffold exposure and infection, with partial/complete loss of the graft) and late complications (i.e., complications that occurred* after* the placement of the implant, such as late graft dehiscence/exposure and infection, peri-implant mucositis, peri-implantitis, and any peri-implant bone loss without signs of infection). With regard to late biological complications, peri-implant mucositis was defined as an inflammation of the soft tissues around the implant, with pain/discomfort and swelling, but in absence of peri-implant bone loss [27]. Peri-implantitis was defined as a condition in which pain, suppuration, exudation, and fistula formation were present, with peri-implant marginal bone loss >2.5 mm and probing pocket depth ≥6 mm [27]. Peri-apical radiographs were taken, at different follow-up sessions, in order to evaluate the presence of any radiolucency around the fixtures.

### 2.7. Statistical Evaluation

Patient demographics and distribution of implants were analysed using descriptive statistics. Means and standard deviations, ranges, and confidence intervals (95%) were calculated for quantitative variables, such as patient age, and gain in vertical and horizontal dimensions of the alveolar ridge. Absolute and relative frequency distributions were calculated for qualitative variables, both patient-related (patient gender, smoking habit) and implant-related (implant site and position, type of prosthetic restoration). The incidence of early and late biological complications (pain/discomfort and edema/swelling after surgery, early/late scaffold exposures and/or infection, partial/complete graft loss, peri-implant mucositis, peri-implantitis, peri-implant bone loss in absence of clinical signs of infection) as well as the implant survival rate was calculated and expressed as percentages. All computations were carried out inside a dedicated datasheet (Excel 2003; Microsoft, Redmond, WA, USA).

## 3. Results

Fifteen patients (6 males and 9 females; aged between 48 and 65 years, mean age 54.2 ± 5.5 years, median 55, confidence interval 95%: 51.5–56.9) were selected for the present clinical retrospective study. Among these, four were smokers (4/15: 26.6%). All patients had been treated with a custom-made synthetic scaffold over a 7-year period, between January 2007 and January 2014; therefore the follow-up varied from 2 to 8 years (with a mean follow-up time of 4.7 years). In all patients, the regenerative surgical procedure went well. In fact, the custom-made synthetic scaffolds perfectly fitted in the bone anatomy and were therefore easily adapted to the bone defects during surgery, secured by titanium mini-screws. This excellent matching of the size/shape helped the surgeon to reduce the operation time. The healing period was uneventful for 10 patients. Five patients, however, experienced early biological complications. In fact, two of these patients (2/15: 13.3%) had mild pain and slight edema/swelling in the first week after surgery. These light symptoms/signs disappeared within two weeks. However, in the other three patients (3/15: 20.0%), early exposure of the custom-made synthetic bone graft occurred, 1, 3, and 5 months after the reconstructive surgery, respectively. These early exposures forced the surgeon to open a new full-thickness flap and to remove part of the synthetic scaffold, with the aid of a piezo-electric device. The surface of the graft was carefully cleaned, and the flap was sutured over it. All these patients were asked to apply 1% chlorhexidine gel, 2 times per day, over the site and were instructed to rinse with 0.12% chlorhexidine, 3 times per day, for a period of 1 week. After this treatment, two of the exposures were resolved with complete reepithelization of the areas and soft tissue closure, in a period of between 2-3 weeks: these early exposures did not prevent proper graft incorporation and consolidation into native bone. However, one of the exposures (1/15: 6.6%) could not be solved and determined the infection and complete loss of the graft, 5 months after surgery, in a 59-year old male smoking patient. In all the 14 remaining patients, after the 8-month healing period, a newly formed, well-incorporated bone was observed, completely filling the bony defects and therefore allowing the placement of an implant in the proper position. After 8 months, the CBCT evaluation revealed a mean vertical bone gain of 2.1 ± 0.9 mm (range 0–3.3 mm, median 2.4 mm, CI 95%: 1.7–2.5 mm) combined with a mean horizontal bone gain of 3.0 ± 1.0 mm (range 0–4.5 mm, median 3.2 mm, CI 95%: 2.5–3.5 mm). In total, 14 implants were successfully placed (6 in the anterior maxilla, 4 in the posterior maxilla, and 4 in the anterior mandible). All these implants (14/14: 100%) were restored with single crowns. No further (late) biological complications were reported, and an implant survival rate of 100% (14/14 surviving implants) was found.

## 4. Discussion

The use of implants for supporting dental prostheses is continuously expanding and it is estimated that the market will have the same trend in the future [1–3].

One of the main limitations of the implant treatment is the unavailability of adequate bone support, mainly caused by periodontal disease, but also as a result of tooth agenesis, traumatic injuries, or other lesions (cysts, tumors) [4, 5].

Many different surgical reconstructive techniques have been introduced in order to reestablish an adequate bone volume and allow proper implant placement [9–14]: among these, alveolar ridge augmentation by means of onlay/inlay bone blocks [10, 11], as well as GBR [9, 28], maxillary sinus elevation [12, 29], and split-crest [14] are the most popular.

Autogenous bone is still considered the material of choice in bone augmentation procedures [15]. However, the use of this material has disadvantages: the need for an additional surgical site, the more invasive procedure, the quantitative limit of bone that can be harvested from the donor site, and the morbidity for the patient [4, 16, 30]. For this reason, different materials have been proposed as possible alternatives, such as allografts [31] and xenografts [32]. Although both allografts and xenografts have been extensively used in bone reconstruction procedures [31, 32], the use of these materials will probably be restricted in future, because they may carry the risk of disease transmission: the processes for their preparation and sterilization might not totally exclude the presence of active viruses or prions [33, 34].

More recently, synthetic bone grafts (alloplasts) have been introduced, in order to overcome these limitations [19, 33–35]. The fundamental properties that a synthetic material should possess are biocompatibility, bioresorbability, and the presence of an architecture/structure similar to that of natural bone: the internal geometry of the biomaterial is, in fact, crucial for the biological behaviour and for promoting new bone formation [16–19, 35]. The modern synthetic porous scaffolds possess a controlled, high porosity and they have a honeycomb structure with several interconnections between different pores [16–19]. This peculiar architecture has proven to be able to stimulate differentiation of mesenchymal cells into functional osteoblasts and finally to promote new bone apposition [16–19]. In addition, the apatite porous spaces and concavities may represent a good microenvironment for angiogenesis, which is fundamental to bring cells and soluble signals like growth factors, and to sustain the regenerative process; angiogenesis is a prerequisite for osteogenesis [16–19].

Blocks of synthetic biomaterials are already available in the market. However, these blocks are prepared in preformed size/shapes and need to be adapted to the patient's bone defect during the surgery [20, 21, 23, 24]. The manual preparation of the required size/shape and the adaptation of these blocks to the bone defect are difficult for the surgeon and may lead to various risks such as the unsatisfactory stabilization/integration of the biomaterial block with the native bone, mobility, and failure of the entire procedure [20, 21, 23, 24]. Moreover, the manual adaptation of the graft greatly increases the time of surgery [23, 24].

Nowadays, modern digital technologies allow the surgeon to virtually design and then fabricate custom-made synthetic porous scaffolds, for use in bone reconstructive procedures [20, 23–26, 36]. In different medical fields, several studies have demonstrated that the combination of modern image acquisition techniques with 3D reconstruction software allows the clinician to obtain custom-made scaffolds for the regeneration of bone structures [20, 23–26, 36–40]. This powerful combination allows the surgeon to virtually plan the reconstruction of an atrophic bone area on his/her computer and to fabricate a custom-made biocompatible scaffold designing its size, thickness, and shape [20, 23–26, 36–40]. The fabrication of the scaffold can be obtained by milling blocks of synthetic bone substitutes that mimic the structure of natural bone and therefore promote the formation of new bone when implanted in the area of defect [36–40].

The CAD/CAM procedure for the fabrication and application of custom-made synthetic scaffolds can be divided into three different steps: the virtual planning and design of the scaffold, the milling of the scaffold into the exact size/shape from a preformed synthetic bone block, and, finally, the reconstructive surgery [20, 21, 24, 26]. The first step starts with a CBCT scan of the interested jaw and the upload of scan data into a 3D reconstruction software [20, 21]. This dedicated software allows the surgeon to analyse the defect area; with the aid of another reverse-engineering software, the virtual reconstruction is finalised [20, 21, 24, 26]. The second step is to transfer the files of the virtual scaffold into a milling machine, where the fabrication process starts, from a preformed standardized synthetic bone block [20, 21, 24, 26]. As soon as the custom-made scaffold is ready, it is sterilised and finally delivered to the surgeon for the clinical application [24, 26].

Jacotti et al. [22] reported on the reconstruction of the atrophic right posterior mandible of a 48-year-old woman, using a dehydrated homologous bone block, shaped with a CAD/CAM technique. The CAD/CAM technique was aimed at avoiding the harvesting of autologous bone block and at assuring a perfect fitting of the block above the alveolar crest [22]. The CAD/CAM technique was successful, with an horizontal bone gain of 6.0, 7.3, and 8.0 mm (mean, 7.18 mm) at sites 6, 12, and 18 mm posterior to the right mental foramen, respectively, 7 months after the reconstructive surgery [22]. Similar results were reported by Figliuzzi et al. [21] for reconstruction of vertical bone defects of the posterior mandible. In this clinical research article, the accuracy of the CAD/CAM scaffolds helped to reduce the time for the operation and contributed to the good healing of the defects; in fact, 6 months after the surgery, a newly formed and well-integrated bone was observed, completely filling the mandibular posterior defects [21]. Accordingly, implants were placed with good primary stability [21]. After 1 year of function, the implant-supported restorations showed no complication, with an excellent biological and esthetic integration [21]. In a case report and review of the literature, Garagiola et al. [36] confirmed the time efficiency and reliability of these CAD/CAM procedures.

In our present study, we have reported on the clinical and radiographic outcomes obtained with this innovative, CAD/CAM procedure for alveolar ridge augmentation. From a surgical point of view, the custom-made synthetic scaffolds were of satisfactory size, shape, and appearance; they matched the defect area, suited the surgeon's requirements, and were easily implanted. This perfect match contributed to reducing the time for surgery and to the good healing of the bone defect. Only a limited number of patients experienced biological complications, such as pain/swelling (2/15: 13.3%) and exposure of the scaffold (3/15: 20.0%); one of these patients, however, experienced infection of the scaffold and complete graft loss. In all other patients, 8 months after reconstruction, a well-integrated newly formed bone was clinically available, and the CBCT evaluation revealed a mean vertical and horizontal bone gain of 2.1 ± 0.9 mm and 3.0 ± 1.0 mm, respectively. Fourteen implants were placed and restored with single crowns. The implant survival rate was 100%.

The present CAD/CAM technique for the fabrication of custom-made synthetic scaffolds for alveolar ridge reconstruction undoubtedly has varied benefits: in fact, the accurate reproduction of the patient's anatomy helps to reduce the time needed for the surgical procedure and therefore the morbidity and risk of infection for the patient [20, 21, 24, 26]. In addition, the increased stability of the bone block may contribute to faster and better bone healing and graft incorporation/consolidation [20, 21, 24, 26]. No gaps were evidenced between the custom-made synthetic scaffolds and the natural bone during the surgery.

However, this procedure has limitations. As reported by the current literature, for a successful alveolar ridge augmentation it is necessary to achieve a perfect fit of the bone block, a precise stabilization of the graft but also a well vascularized bone bed [20, 21]. The custom-made scaffolds can certainly help to obtain an excellent fit and stability of the graft; however, they must be limited in dimensions, to allow for proper cellular and vascular penetration [20, 21, 24]. If the graft is too big, in fact, the vascular penetration cannot be completed and there is the possibility of early or late graft exposure, with high risk of infection of the graft: in this sense, there is no difference between the present CAD/CAM technique and the more conventional techniques using onlay grafts for alveolar ridge augmentation. In the present study, the graft bed had been prepared using small perforations, 1-2 mm deep, on the bony walls, in order to increase the amount of bleeding. It is clear that the larger the graft is, the more difficult it is for cells and vessels to colonize it. In the present study, we did not treat defects wider than 12 mm in height and 10 mm in width; in addition, patients had 3-wall bone defects. Despite this, the present procedure presented a rather high percentage of biological complications, such as early graft exposure (20%). The exposure of the synthetic scaffold must be considered an adverse event and a difficult complication to manage: in fact, it can lead to partial or complete loss of the graft [20, 21]. In the last few years, several synthetic scaffolds with different macro- and microporosity and geometry have been introduced [16–19, 35]. These materials can certainly improve the healing processes; however, the perfect characteristics for a synthetic porous scaffold still need to be elucidated. When future innovations provide the possibility to seed customized scaffolds with components such as growth factors and stem cells, the indication of this procedure might be extended to bigger defects [26, 37, 39]. Another limitation of the present surgical technique for alveolar ridge augmentation is that it requires a high level of surgical skill, particularly with regard to the ability to properly treat soft tissues [20, 26]. Once again, in this sense, there is no difference compared to the more conventional techniques. In fact, a tension-free primary closure of the flap is essential, in order to avoid exposure of the synthetic scaffold. An early (or late) exposure may, in fact, jeopardize the success of the regenerative procedure [20, 26]. Lastly, the final limitation of the present CAD/CAM technique is related to the presence of metal crowns or amalgam restorations close to the area to be reconstructed [20, 26]. When the images from the CBCT are inserted in the software for the treatment planning, the metal artifacts might not allow the clinician to clearly identify the margins of the bone defect: this may potentially lead to an inappropriate design of the scaffold and consequently to a poor clinical adaptation.

Although the procedure for the design, fabrication, and clinical application of custom-made synthetic bone grafts described here presents the aforementioned limitations, our present positive clinical and radiographic outcomes seem to suggest it as a possible alternative to conventional surgical techniques, such as alveolar ridge augmentation with onlay/inlay autogenous bone blocks [20, 21, 26]. It is important, however, to point out the inherent limits of our present study. In fact, it is retrospective in design and the conclusions are based on a limited number of patients (15). Further studies with a larger patient sample and with a more appropriate design (prospective controlled studies or even better, randomized controlled trials) are needed to confirm the positive outcomes emerging from our investigation.

## 5. Conclusions

In the present retrospective clinical study, we have presented an innovative CAD/CAM technique for the design, fabrication, and clinical application of custom-made synthetic bone grafts, for alveolar ridge augmentation. Although positive clinical and radiographic outcomes have been found in this study, with an excellent fit of the scaffolds during surgery and a well-integrated newly formed bone clinically available 8 months after bone reconstruction, a rather high incidence of biological complications, such as early graft exposure (20%), were reported. Further studies with a larger patient sample and an appropriate design (such as prospective studies or randomized controlled trials) are therefore needed to draw specific conclusions about the reliability of the present technique and to confirm our positive clinical and radiographic outcomes.
